# Cationic Ordering and Its Influence on the Magnetic Properties of Co-Rich Cobalt Ferrite Thin Films Prepared by Reactive Solid Phase Epitaxy on Nb-Doped SrTiO_3_(001)

**DOI:** 10.3390/ma15010046

**Published:** 2021-12-22

**Authors:** Jannis Thien, Jascha Bahlmann, Andreas Alexander, Kevin Ruwisch, Jari Rodewald, Tobias Pohlmann, Martin Hoppe, Fatih Alarslan, Martin Steinhart, Baki Altuncevahir, Padraic Shafer, Carola Meyer, Florian Bertram, Joachim Wollschläger, Karsten Küpper

**Affiliations:** 1 Department of Physics, Osnabrück University, 49076 Osnabrück, Germany; jthien@uos.de (J.T.); jbahlmann@uos.de (J.B.); aalexander@uos.de (A.A.); kruwisch@uos.de (K.R.); jarodewa@uos.de (J.R.); tobias.pohlmann@uos.de (T.P.); mahoppe@uos.de (M.H.); carola.meyer@uos.de (C.M.); jwollsch@uos.de (J.W.); 2 Deutsches Elektronen-Synchrotron (DESY), Photon Science, 22607 Hamburg, Germany; florian.bertram@desy.de; 3 Institute for Chemistry of New Materials and Center for Cellular Nanoanalytics (CellNanOs), Osnabrück University, 49076 Osnabrück, Germany; falarslan@uos.de (F.A.); martin.steinhart@uos.de (M.S.); 4 Physics Engineering Department, Faculty of Science and Letters, Istanbul Technical University, Maslak, Istanbul TR-34469, Turkey; altuncevahir@itu.edu.tr; 5 Advanced Light Source, Lawrence Berkeley National Laboratory, Berkeley, CA 94720, USA; pshafer@lbl.gov

**Keywords:** cobalt ferrite, ultrathin films, magnetic properties, cationic distribution

## Abstract

Here, we present the (element-specific) magnetic properties and cation ordering for ultrathin Co-rich cobalt ferrite films. Two Co-rich ${\mathrm{Co}}_{x}{\mathrm{Fe}}_{3-x}O_{4}$ films with different stoichiometry (x=1.1 and x=1.4) have been formed by reactive solid phase epitaxy due to post-deposition annealing from epitaxial CoO/Fe${}_{3}$O${}_{4}$ bilayers deposited before on Nb-doped SrTiO${}_{3}$(001). The electronic structure, stoichiometry and homogeneity of the cation distribution of the resulting cobalt ferrite films were verified by angle-resolved hard X-ray photoelectron spectroscopy. From X-ray magnetic circular dichroism measurements, the occupancies of the different sublattices were determined using charge-transfer multiplet calculations. For both ferrite films, a partially inverse spinel structure is found with increased amount of ${\mathrm{Co}}^{3+}$ cations in the low-spin state on octahedral sites for the ${\mathrm{Co}}_{1.4}{\mathrm{Fe}}_{1.6}O_{4}$ film. These findings concur with the results obtained by superconducting quantum interference device measurements. Further, the latter measurements revealed the presence of an additional soft magnetic phase probably due to cobalt ferrite islands emerging from the surface, as suggested by atomic force microscope measurements.

## 1. Introduction

Ferrites such as CoFe${}_{2}$O${}_{4}$ (CFO) have intriguing electronic and magnetic properties that are increasingly attracting attention, particularly for advancing the fields of spintronics and spin caloritronics. For instance, in these fields magnetic insulators (MI) can be used as spin-filters for the generation of highly spin-polarized electron currents due to their spin-dependent band gaps [1,2,3,4,5,6], thereby creating the possibility of faster and less energy consuming spintronic devices. As the spin-filter effect decreases drastically with the thickness of the spin-filter film, ultrathin MI films are essential to realize high-efficiency spin-filters. Additionally, thin CFO films are useful as supports for Pt films to create thermally generated spin currents [7,8] via the spin Seebeck effect (SSE) [9]. Owing to the absence of a magnetic proximity effect in Pt/CoFe${}_{2}$O${}_{4}$ bilayers [10], no parasitic effects, such as the anomalous Nernst effect [11], are induced, simplifying the evaluation of the SSE signal and thus making CFO films also quite interesting for spin caloritronic applications. Furthermore, CFO has significant potential in the fields of iron-based supercapacitors [12] and electrocatalysts [13].

Whereas stochiometric CoFe${}_{2}$O${}_{4}$ (x=1) and the Co-deficient phase ${\mathrm{Co}}_{x}{\mathrm{Fe}}_{3-x}O_{4}$ (x<1) have been investigated intensely during the last two decades, the Co-rich phase ${\mathrm{Co}}_{x}{\mathrm{Fe}}_{3-x}O_{4}$ (x>1) has gained only little attention up to now. Nevertheless, Co${}_{2}$FeO${}_{4}$ thin films have been reported in one of these few works to have highly interesting magnetic properties with the potential for novel spintronic applications [14].

Stoichiometric CFO crystallizes as a cubic inverse spinel with a lattice constant of 8.392 Å. For a perfect inversion of the spinel structure, the octahedral *B* lattice sites in the face-centered cubic oxygen sublattice of CFO are occupied by divalent ${\mathrm{Co}}^{2+}$ cations, while trivalent ${\mathrm{Fe}}^{3+}$ cations occupy both the octahedral *B* lattice sites and the tetrahedral *A* lattice sites with a 1:1 distribution. Due to the antiferromagnetic super-exchange interaction between the ${\mathrm{Fe}}^{3+}$ cations at the *A* and *B* lattice sites, their magnetic moments effectively compensate for each other. Hence, the resulting magnetization originates mostly from the ferromagnetic interaction between the ${\mathrm{Co}}^{2+}$ and ${\mathrm{Fe}}^{3+}$ cations at the *B* lattice sites, resulting in a net magnetization of 3 $\mu_{B}/f.u.$ [1] due to the spin moment of ${\mathrm{Co}}^{2+}$ and neglecting orbital moments.

However, it has been reported that cobalt ferrite quite often exhibits only partially inverse spinel structure [1,15] with ${\mathrm{Co}}^{2+}$ on both tetrahedral *A* and octahedral *B* lattice sites. As a consequence, the distribution of cations can have a strong impact on the magnetic properties and thus also on the resulting net magnetization based on the magnitude of the cation disorder. In principle, the cation disorder of stoichiometric CoFe${}_{2}$O${}_{4}$ can be described by the inversion parameter γ with 0≤γ≤1 according to the structural formula

(1) $${\left[{\mathrm{Co}}_{1-\gamma }^{2+}{\mathrm{Fe}}_{\gamma }^{3+}\right]}_{A}{\left[{\mathrm{Co}}_{\gamma }^{2+}{\mathrm{Fe}}_{2-\gamma }^{3+}\right]}_{B}O_{4}^{2-}.$$

The indices *A* and *B* represent the respective lattice sites. For γ=0, all ${\mathrm{Co}}^{2+}$ cations occupy tetrahedral *A* lattice sites and all ${\mathrm{Fe}}^{3+}$ cations occupy octahedral *B* lattice sites, corresponding to the case for a normal spinel structure. In contrast, γ=1 indicates a complete inverse spinel. Values between γ=0 and γ=1 characterize intermediate states with a partially inverse cation distribution.

As for the Co-rich phase, there have been contradicting results for the coordination of the excessive Co ions. Whereas Mössbauer spectroscopy studies suggest ${\mathrm{Co}}^{3+}$ cations in octahedral coordination, which are in the low-spin state [16], more recent work found ${\mathrm{Co}}^{3+}$ cations in tetrahedral coordination and in high-spin state [17]. We wanted to go a step further and performed a comprehensive analysis of the electronic and magnetic properties of ultrathin Co ferrite films, which we produced very recently by intermixing epitaxial CoO/Fe${}_{3}$O${}_{4}$ bilayers using a post-deposition annealing (PDA) approach [18].

Generally, cobalt ferrite thin films are often prepared by deposition methods such as molecular beam epitaxy [19], sputter deposition [5] and pulsed laser deposition [20]. Quite recently, we demonstrated that thin cobalt ferrite films can be prepared by reactive solid phase epitaxy (RSPE) due to the intermixing of epitaxial Fe${}_{3}$O${}_{4}$/CoO and CoO/Fe${}_{3}$O${}_{4}$ bilayers [18,21]. For both bilayer systems, the intermixing of the oxide layers was caused by PDA on Nb-doped SrTiO${}_{3}$(001) substrates (lattice constant 3.905 Å). However, the large lattice mismatch of 7.45% between the formed Co ferrite film and the SrTiO${}_{3}$ (comparing the lattice constant of SrTiO${}_{3}$ with the halved lattice constant of CFO) induces strain in the Co ferrite films. This strain, on the other hand, may also be used to steer the physical properties of these Co ferrite films [22,23], especially their electronic and magnetic properties, as demonstrated, for example, in Fe${}_{3}$O${}_{4}$ thin films prepared on SrTiO${}_{3}$(001) [24,25].

Since the cation distribution of cobalt ferrite can easily be changed by thermal treatment [26,27], we report here on a case study concerning mainly the cation distribution and the resulting magnetic behavior of two cobalt ferrite films prepared by RSPE. To gain insight into the occupation of the octahedral and tetrahedral sites in the inverse spinel structure and to probe the element-specific magnetic properties of the cobalt ferrite films, X-ray absorption spectroscopy (XAS) and X-ray magnetic circular dichroism (XMCD) measurements were utilized. The integral magnetic properties were examined by superconducting quantum interference device (SQUID) measurements. Furthermore, the electronic and chemical properties throughout the whole films and the surface morphologies of the films were studied by angle-resolved hard X-ray photoelectron spectroscopy (AR-HAXPES) and atomic force microscope (AFM) measurements, respectively. Detailed knowledge of the cationic distribution is a key point in understanding the complex magnetic properties of these cobalt ferrites, which is in turn necessary for potential future applications mentioned above.

## 2. Materials and Methods

**Figure 1:**
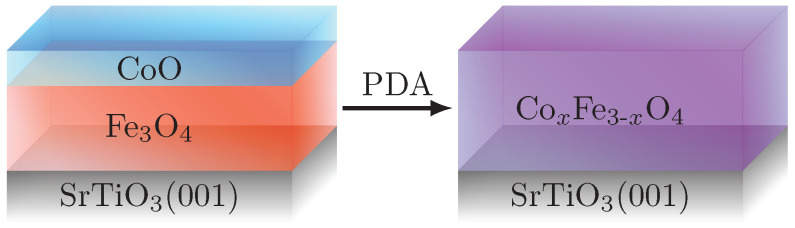
Principal sketch of the conducted film preparation. The thermally induced interdiffusion results in the formation of homogeneous ${\mathrm{Co}}_{x}{\mathrm{Fe}}_{3-x}O_{4}$ films from initial epitaxial CoO/Fe${}_{3}$O${}_{4}$ bilayers grown on Nb-doped SrTiO${}_{3}$(001).

Two ${\mathrm{Co}}_{x}{\mathrm{Fe}}_{3-x}O_{4}$ films with stoichiometries of x=1.1 and x=1.4 and total film thicknesses of 16.5±0.5 nm and 18.1±0.5 nm, respectively, were prepared by RSPE from epitaxial CoO/Fe${}_{3}$O${}_{4}$ bilayers grown on Nb-doped SrTiO${}_{3}$(001) substrates (cf. Figure 1) at the beamline BM25-SpLine of the European Synchrotron Radiation Facility (ESRF) [18]. The sample preparation was extensively monitored by several chemical and structural characterization techniques such as soft and hard X-ray photoelectron spectroscopy and synchrotron radiation based X-ray diffraction including surface sensitive grazing incidence X-ray diffraction to follow the evolution from the CoO/Fe${}_{3}$O${}_{4}$ bilayer stacks to completely reacted Co ferrite films. A detailed description of the sample preparation can be found in reference [18].

To probe the homogeneity of the cation distribution as well as the electronic and chemical properties after the whole PDA treatment, AR-HAXPES measurements at beamline P22 of PETRA III at Deutsches Elektronen-Synchrotron (DESY) were conducted using an excitation energy of hν=5945 eV. The photoelectrons were detected by a Phoibos 225 HV hemispherical analyzer (SPECS, Berlin, Germany) with a delay-line electron detector. As a result of the finite acceptance angle of the detector, increasing the glancing incidence angle of the X-ray beam with respect to the sample surface leads to a higher surface sensitivity since less photoelectrons from the bulk are detected due to the fixed angle between source and detector. Consequently, lower glancing incidence angles allow probing of deeper layers of samples and vice versa. In order to probe both the bulk and the surface-near region of the formed ${\mathrm{Co}}_{x}{\mathrm{Fe}}_{3-x}O_{4}$ films, incidence angles of 5°, 45°, and 60° were used.

The surface morphologies of the resulting ${\mathrm{Co}}_{x}{\mathrm{Fe}}_{3-x}O_{4}$ films were examined by AFM measurements using a NT-MDT NTEGRA device. The measurements were performed in contact mode. For the measurements, a field of view area of 1500 nm×1000 nm was chosen.

XAS and XMCD measurements at the Co $L_{2,3}$ (770–818 eV) and Fe $L_{2,3}$ (700–750 eV) edges were conducted at beamline 4.0.2 of the Advanced Light Source (ALS) using an external magnetic field of 4 T parallel to the X-ray beam and a degree of circular polarization of 90%. For each $L_{2,3}$ edge, two absorption spectra were recorded for two opposite directions of the external magnetic field, which is analogous to changing the helicity of the circular polarization. Each absorption spectrum was recorded utilizing the total electron yield (TEY). From the sum and the difference of the two absorption spectra, the corresponding XAS and XMCD signals were obtained, respectively. All XAS and XMCD measurements were carried out at a temperature of 300 K and at a glancing incidence angle of 30° between the surface of the samples and the X-ray beam.

The XA and XMCD spectra were analyzed according to full multiplet calculations based on crystal-field theory and charge-transfer [28] using CTM4XAS [29]. In addition, we used a spin–orbit coupling of 100% and the Slater integrals F(dd), F(pd), and G(pd) to consider *d*–*d* and *p*–*d* Coulomb and exchange interactions [30]. To take into account interatomic screening, the Slater integrals were reduced to 80% of their atomic values. Furthermore, all transition lines at the $L_{3}$($L_{2}$) edges were broadened by a Lorentzian width of 0.25 eV ( 0.45 eV) due to core–hole lifetime broadening and by a Gaussian width of 0.2 eV due to instrumental broadening.

SQUID measurements were performed at the Diamond Light Source (DLS) to get better insight on the integral magnetic properties of the samples. Magnetization curves from −5 T to 5 T were recorded at 300 K and 5 K using a MPMS system (MPMS3, Quantum Design, Darmstadt, Germany). The magnetization curves were corrected with respect to all non-ferrimagnetic contributions, such as the diamagnetic background caused by the SrTiO${}_{3}$ substrates and the sample holder.

## 3. Results and Discussion

### 3.1. AR-HAXPES

**Figure 2:**
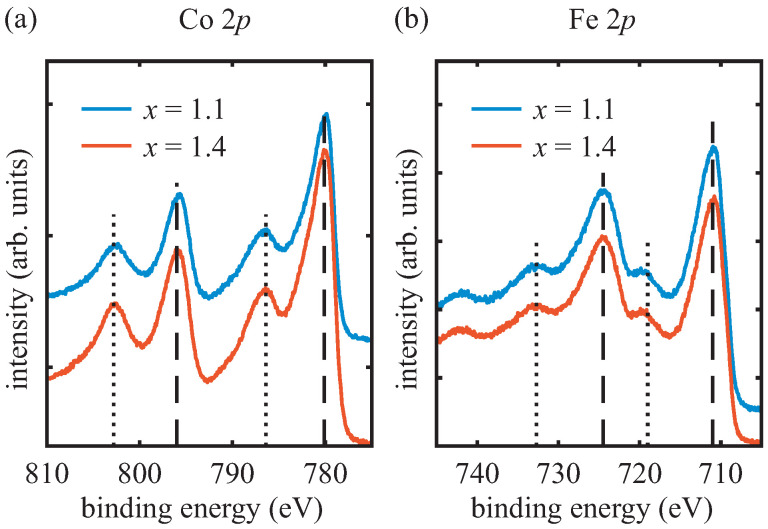
Angular integrated (**a**) Co 2p and (**b**) Fe 2p HAXPES spectra for both samples. In both spectra, the dashed lines indicate positions of the particular $2p_{1/2}$ and $2p_{3/2}$ peaks. The dotted lines correspond to positions of the shake-up satellites in (**a**) and the charge-transfer satellites in (**b**).

In Figure 2, the Co 2p and Fe 2p HAXPES spectra (angular integrated) of each sample are displayed. All recorded spectra were calibrated according to the O 1s core level at 530 eV binding energy.

The Co 2p spectra show the characteristic shape for cobalt ferrite [19,31]. The main peaks ($2p_{1/2}$ and $2p_{3/2}$) are located at binding energies of 795.8±0.3 eV and 780.1±0.3 eV, respectively. The peaks are accompanied by one shake-up satellite each, lying about 6 eV at higher binding energies.

For the Fe 2p spectra, the Fe $2p_{1/2}$ and Fe $2p_{3/2}$ peaks have binding energies of (724.5±0.3) eV and (711.0±0.3) eV, respectively. In addition, the Fe 2p spectrum of each sample exhibits distinct charge-transfer satellites at (719.0±0.4) eV and (732.7±0.4) eV. Both the appearance of the charge-transfer satellites and the positions of the Fe 2p peaks indicate a majority of ${\mathrm{Fe}}^{3+}$ cations [32,33,34], as expected for cobalt ferrite [19].

In order to gain information on the chemical composition and the homogeneity of the Co and Fe cation distribution of the cobalt ferrite films, a quantitative analysis was performed for each incidence angle mentioned in the experimental details section [18]. We found for each cobalt ferrite film a homogeneous depth distribution of the Co and Fe cations throughout the whole film. Thus, both oxide films are fully intermixed, confirming the formation of single ${\mathrm{Co}}_{x}{\mathrm{Fe}}_{3-x}O_{4}$ films with stoichiometries of x=1.1 and x=1.4. Nevertheless, an additional rock-salt phase was observed for the ${\mathrm{Co}}_{1.4}{\mathrm{Fe}}_{1.6}O_{4}$ film. This additional phase coexists homogeneously distributed with the ferrite spinel phase, with both phases together forming a single film rather than a bilayer structure. For more details, see reference [18].

### 3.2. AFM

**Figure 3:**
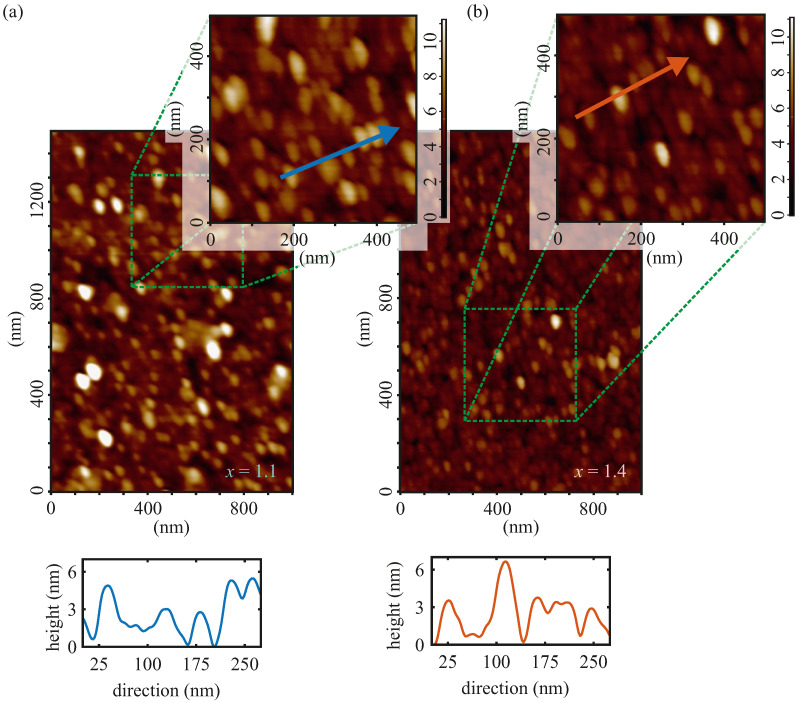
Representative AFM images and height profiles of the ${\mathrm{Co}}_{x}{\mathrm{Fe}}_{3-x}O_{4}$ films with (**a**) x=1.1 and (**b**) x=1.4. The blue and red arrows represent the directions of the respective height profiles presented underneath.

The morphologies of the cobalt ferrite film surfaces of both samples were obtained by AFM and are depicted in Figure 3. An area of 1500 nm×1000 nm was used for the AFM micrographs in both cases. Respective height profiles were made to estimate the average island heights and sizes.

For both samples, the cobalt ferrite film surface is covered by islands with an average island size of 55±5 nm in diameter. Similar results of CoFe${}_{2}$O${}_{4}$ thin films prepared by radiofrequency magnetron sputtering on SrTiO${}_{3}$(001) were obtained by Rigato et al. [35]. The average island heights of both films are comparable (cf. respective height profiles), although the ${\mathrm{Co}}_{x}{\mathrm{Fe}}_{3-x}O_{4}$ film with x=1.1 has occasionally even significantly higher islands. A root mean square roughness (RMS) analysis revealed vertical RMS roughnesses of (1.7±0.2) nm and (1.3±0.2) nm for the ${\mathrm{Co}}_{1.1}{\mathrm{Fe}}_{1.9}O_{4}$ film and the ${\mathrm{Co}}_{1.4}{\mathrm{Fe}}_{1.6}O_{4}$ film, respectively. Thus, the ${\mathrm{Co}}_{1.4}{\mathrm{Fe}}_{1.6}O_{4}$ film has a lower vertical roughness than the ${\mathrm{Co}}_{1.1}{\mathrm{Fe}}_{1.9}O_{4}$ film. It should be noted that the sporadically occurring higher islands on the ${\mathrm{Co}}_{1.1}{\mathrm{Fe}}_{1.9}O_{4}$ film’s surface were not included in the RMS analysis. Hence, the volume fraction of the islands relative to the total film volume is about 15% (at least) for the ${\mathrm{Co}}_{1.1}{\mathrm{Fe}}_{1.9}O_{4}$ film and about 10% for the ${\mathrm{Co}}_{1.4}{\mathrm{Fe}}_{1.6}O_{4}$ film.

### 3.3. XAS/XMCD

**Figure 4:**
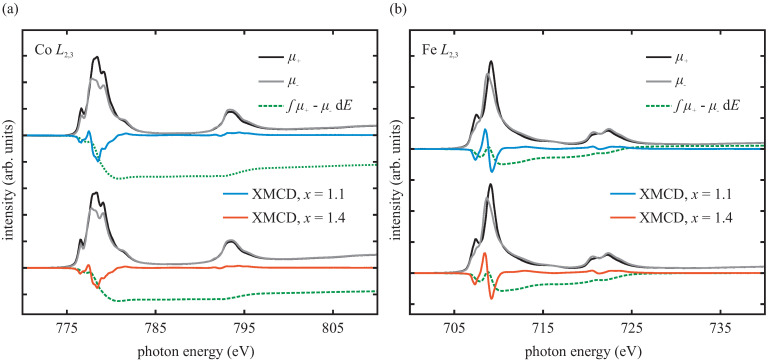
XMCD spectra (blue and red) and integrated XMCD spectra (dashed green) recorded at 300 K across the (**a**) Co $L_{2,3}$ and (**b**) Fe $L_{2,3}$ edges of both samples. The spectra in gray ($\mu_{+}$) and black ($\mu_{-}$) are the absorption spectra recorded with two opposite directions of the external magnetic field.

To probe the cationic distribution of the resulting cobalt ferrite films after the heat treatment, and to gain element-specific information about the resulting magnetic properties, we performed XAS/XMCD measurements at the Co $L_{2,3}$ and Fe $L_{2,3}$ edges. The absorption spectra with their resulting XMCD spectra at 300 K of both samples are depicted in Figure 4.

The absorption spectra at the Co $L_{2,3}$ edges of both samples exhibit the characteristic shape of predominant divalent Co [21,36,37]. For the Fe $L_{2,3}$ edges, the shape of the absorption spectra of both samples resembles the shape of the Fe $L_{2,3}$ spectra of CoFe${}_{2}$O${}_{4}$ and Fe${}_{2}$O${}_{3}$ with predominant trivalent Fe [21,38,39,40].

Further, both samples show strong magnetic dichroic signals of Co and Fe. Compared to the ${\mathrm{Co}}_{x}{\mathrm{Fe}}_{3-x}O_{4}$ film with x=1.4, the ${\mathrm{Co}}_{x}{\mathrm{Fe}}_{3-x}O_{4}$ film with x=1.1 exhibits an increased Co XMCD signal, indicating a higher magnetic moment of Co. The Fe XMCD signals are almost commensurate.

From the Co XMCD and Fe XMCD spectra, the orbital moment $m_{o}$ and the spin moment $m_{s}$ of the Co and Fe ions were determined, by applying the sum rules [41,42,43,44] and the sum rules’ correction factors as derived by Teramura et al. [45]. The correction factors take into account the mixing of the $L_{2}$ and $L_{3}$ excitations due to core–hole interactions [45]. The values of the orbital moment $m_{o}$, the spin moment $m_{s}$, and the resulting total moments of the respective Co and Fe cations are listed in Table 1 for both Co ferrite films. The results reveal for the ${\mathrm{Co}}_{1.1}{\mathrm{Fe}}_{1.9}O_{4}$ film a significantly higher total Co moment per ion compared to the ${\mathrm{Co}}_{1.4}{\mathrm{Fe}}_{1.6}O_{4}$ film. The total Fe moments per ion of both Co ferrite films are commensurate considering experimental uncertainties, as noted before.

**Figure 5:**
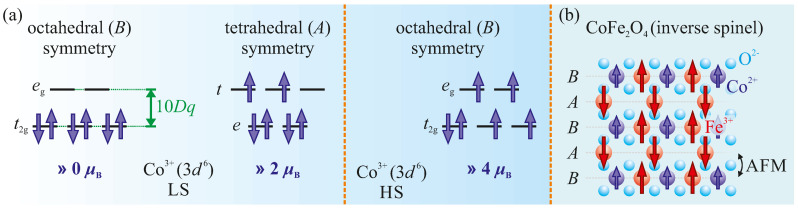
(**a**) The occupation of the 3d states of ${\mathrm{Co}}^{3+}$ in the low-spin (LS) state at either octahedral *B* or tetrahedral *A* lattice sites, including the resulting net spin moments. The occupation of octahedrally coordinated ${\mathrm{Co}}^{3+}$ in the high-spin (HS) state is shown for comparison. (**b**) Antiferromagnetic (AFM) coupling between cations at octahedral *B* and tetrahedral *A* lattice sites for stoichiometric $\mathrm{Co}{\mathrm{Fe}}_{2}O_{4}$ as an inverse spinel.

For the Co-rich phase of ${\mathrm{Co}}_{x}{\mathrm{Fe}}_{3-x}O_{4}$, it has been reported in the literature that increasing the Co content (x>1) results in an increased amount of ${\mathrm{Co}}^{3+}$ cations [16,46,47,48,49], replacing ${\mathrm{Fe}}^{3+}$ cations in the crystal structure due to charge neutrality. In oxides with (inverse) spinel structures, ${\mathrm{Co}}^{3+}$ cations prefer strongly octahedral sites [15,47,50,51,52]. Depending on the crystal field (cf. Figure 5), these ${\mathrm{Co}}^{3+}$ cations in octahedral coordination can either be found in high-spin state (S = 2) or in diamagnetic low-spin state (S = 0) [50]. For the ${\mathrm{Co}}_{1.4}{\mathrm{Fe}}_{1.6}O_{4}$ film in this present study, only an increased amount of ${\mathrm{Co}}^{3+}$ cations at octahedral *B* sites being in the low-spin state can explain the lower total Co moment per Co ion compared to the ${\mathrm{Co}}_{1.1}{\mathrm{Fe}}_{1.9}O_{4}$ film. In contrast, ${\mathrm{Co}}^{3+}$ in high-spin state would increase the spin moment per Co ion (cf. Figure 5) and thus the total magnetic moment per Co ion.

Assuming instead that no ${\mathrm{Co}}^{3+}$ ions are present in the ferrite film, excess ${\mathrm{Co}}^{2+}$ cations have to occupy tetrahedral *A* sites, reducing the degree of inversion. Thus, a decreased total Co moment per ion could essentially be also related to the antiferromagnetic coupling between ${\mathrm{Co}}^{2+}$ cations, occupying tetrahedral *A* and octahedral *B* sites.

A lower degree of inversion would affect the Fe ions likewise, under the assumption that the total number of cations in the oxygen sublattices remains constant. As a consequence, more Fe ions would be at octahedral *B* sites, resulting in a higher total magnetic moment per Fe ion. This behavior, however, is not observed, since the total Fe moments per ion are fairly equal for both stoichiometries, indicating similar degrees of inversion for the films. Thus, this effect can be excluded as the origin for the reduced total magnetic moment of the ${\mathrm{Co}}_{1.4}{\mathrm{Fe}}_{1.6}O_{4}$ film.

Our previous growth study [18] suggested for the ${\mathrm{Co}}_{1.4}{\mathrm{Fe}}_{1.6}O_{4}$ film in particular the existence of a second crystallographic phase, which was attributed to Co–Fe oxide precipitates in the film. These precipitates are not present in the ${\mathrm{Co}}_{1.1}{\mathrm{Fe}}_{1.9}O_{4}$ film. Both CoO and FeO are antiferromagnetic with bulk Néel temperatures of 293 K [53,54] and 198 K [54], respectively. Including finite-size effects, which reduce the critical temperature, it can be assumed that a solid dispersion of CoO and FeO also has a Néel temperature fairly below 300 K, where the XAS/XMCD measurements were carried out. Hence, the Co–Fe oxide precipitates should be paramagnetic and consequently contribute only slightly, if at all, to the XMCD signal. The magnetic moments of soley the Co ferrite phase is thus slightly underestimated when applying the sum rules.

Assuming as worst case scenario that a second Co–Fe oxide phase forms for a Co content of x>1.1 (cf. ${\mathrm{Co}}_{1.1}{\mathrm{Fe}}_{1.9}O_{4}$ film) and considering that both samples initially had equal ${\mathrm{Fe}}_{3}O_{4}$ film thicknesses, the averaged magnetic moment per Co cation would only be reduced by at most (17±4)% for the ${\mathrm{Co}}_{1.4}{\mathrm{Fe}}_{1.6}O_{4}$ film compared with the ${\mathrm{Co}}_{1.1}{\mathrm{Fe}}_{1.9}O_{4}$ film. Thus, the resulting XAS signal from this phase would be too small to produce such a reduction of the magnetic moments. Moreover, it has been reported that doping of, e.g., paramagnetic ZnO or CuO with even small quantities of transition metal elements such as Co, Fe, and Ni, results in an unexpected low ferromagnetic behavior [55,56,57,58]. Furthermore, it has been demonstrated that CoO, FeO, CuO, and ZnO often exhibit defects such as (oxygen) vacancies, which can also lead to a non-negligible ferromagnetic behavior even above their Néel temperatures [59,60,61,62]. Thus, the averaged magnetic moment per Co cation would even be less reduced, comparing the stoichiometry x=1.4 with x=1.1 due to these effects.

In summary, considering all these possibilities, it is more plausible that the significantly lower total Co moment per ion of the ${\mathrm{Co}}_{1.4}{\mathrm{Fe}}_{1.6}O_{4}$ film is primarily caused by rather an increased amount of trivalent low-spin Co cations at octahedral *B* sites.

Additionally, the XA and XMCD spectra were analyzed simultaneously according to charge-transfer multiplet (CTM) calculations. In these calculations, the transitions from the occupied 2p state into the unoccupied 3d state in each transition metal cation located in an oxygen ligand field are calculated, taking into account multiplet effects and charge-transfer interactions. For ${\mathrm{Co}}_{x}{\mathrm{Fe}}_{3-x}O_{4}$, the different transition metal cations can either be octahedrally or tetrahedrally coordinated by the surrounding oxygen anions due to its (inverse) spinel structure and can also be in high-spin state or low-spin state dependent on the crystal field. The respective XA and XMCD spectra were fitted by a weighted linear superposition, consisting of CTM contributions from the corresponding cationic states.

**Figure 6:**
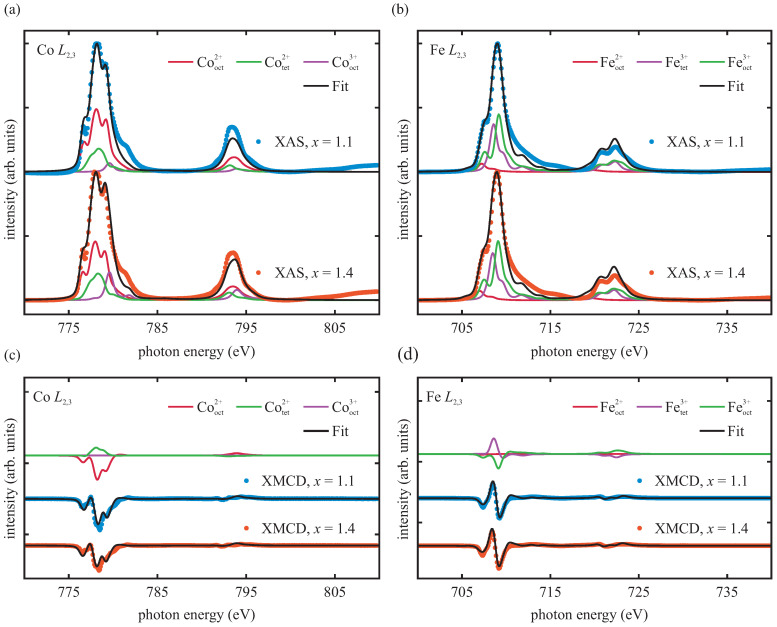
XA and XMCD spectra of the Co $L_{2,3}$ and Fe $L_{2,3}$ edges with their corresponding CTM calculations (black lines) for each sample. The XA and XMCD spectra at the Co $L_{2,3}$ edge in (**a**,**c**), respectively, were fitted with superpositions of octahedral coordinated ${\mathrm{Co}}^{2+}$ and ${\mathrm{Co}}^{3+}$ cations and tetrahedral coordinated ${\mathrm{Co}}^{2+}$ cations. For the XA and XMCD spectra at the Fe $L_{2,3}$ edge in (**b**,**d**), respectively, superpositions of octahedral coordinated ${\mathrm{Fe}}^{2+}$ and ${\mathrm{Fe}}^{3+}$ cations and tetrahedral coordinated ${\mathrm{Fe}}^{3+}$ cations were used. The individual cationic contributions to the total CTM spectra are shown for each XA spectrum and each sample. For the XMCD spectra, only the individual cationic contributions of the ${\mathrm{Co}}_{1.1}{\mathrm{Fe}}_{1.9}O_{4}$ film are depicted for clarity, serving as a representative for the ${\mathrm{Co}}_{1.4}{\mathrm{Fe}}_{1.6}O_{4}$ film.

For the Co $L_{2,3}$ edges, both ${\mathrm{Co}}^{2+}$ and ${\mathrm{Co}}^{3+}$ cations were used to reproduce the data based on a reduced total Co moment per ion originating from low-spin ${\mathrm{Co}}^{3+}$ cations, as suggested before. The ${\mathrm{Co}}^{2+}$ cations were considered at both tetrahedral and octahedral sites with crystal fields of −0.6 eV and 0.8 eV (high-spin state), respectively. Thus, we conclude that the inversion of the spinel structure was not complete. The crystal fields are comparable with values used in previous studies of CoFe${}_{2}$O${}_{4}$ thin films [21]. Since it is reported that ${\mathrm{Co}}^{3+}$ cations preferably occupy octahedral lattice sites [15,47,50,51,52], only ${\mathrm{Co}}^{3+}$ cations in octahedral coordination were assumed for the analysis. Best fits were obtained for ${\mathrm{Co}}^{3+}$ cations in low-spin state with a crystal field of 10 Dq=2.1 eV (cf. Figure 6).

Regarding the Fe $L_{2,3}$ edges, Co ferrite should in principal solely contain Fe cations as ${\mathrm{Fe}}^{3+}$. However, oxygen vacancies or the presence of ${\mathrm{Co}}^{3+}$ cations in the film can lead to the presence of small amounts of ${\mathrm{Fe}}^{2+}$ cations due to preserving charge neutrality of the films [21,63]. In order to account for these effects, ${\mathrm{Fe}}^{2+}$ cations at octahedral sites and ${\mathrm{Fe}}^{3+}$ cations at both tetrahedral and octahedral sites were assumed based on our previous XAS and XMCD results of CoFe${}_{2}$O${}_{4}$[21] and NiFe${}_{2}$O${}_{4}$ [64] thin films. The crystal fields were set to 1.15 eV for ${\mathrm{Fe}}_{\mathrm{oct}}^{2+}$, −0.5 eV for ${\mathrm{Fe}}_{\mathrm{tet}}^{3+}$, and 1.2 eV for ${\mathrm{Fe}}_{\mathrm{oct}}^{3+}$ (high-spin state), which are in good accordance with values used in the literature for both Fe${}_{3}$O${}_{4}$[65,66] and CoFe${}_{2}$O${}_{4}$ [21]. The XA and XMCD spectra of our study with their corresponding best fits are depicted in Figure 6.

The analysis revealed for the ${\mathrm{Co}}_{1.1}{\mathrm{Fe}}_{1.9}O_{4}$ film a cation distribution of

$${\left[{\mathrm{Co}}_{0.25}^{2+}{\mathrm{Fe}}_{0.73}^{3+}\right]}_{A}{\left[{\mathrm{Co}}_{0.80}^{2+}{\mathrm{Fe}}_{0.18}^{2+}{\mathrm{Co}}_{0.05}^{3+}{\mathrm{Fe}}_{0.99}^{3+}\right]}_{B}O_{4-\delta }^{2-}$$

and for the ${\mathrm{Co}}_{1.4}{\mathrm{Fe}}_{1.6}O_{4}$ film a cation distribution of

$${\left[{\mathrm{Co}}_{0.34}^{2+}{\mathrm{Fe}}_{0.59}^{3+}\right]}_{A}{\left[{\mathrm{Co}}_{0.85}^{2+}{\mathrm{Fe}}_{0.16}^{2+}{\mathrm{Co}}_{0.21}^{3+}{\mathrm{Fe}}_{0.85}^{3+}\right]}_{B}O_{4-\delta }^{2-}$$

with δ=0.15±0.25, which is in accordance with the value of δ calculated from relative intensity ratios of the Co 2p, Fe 2p, and O 1s core-level spectra of the AR-HAXPES measurements [18]. We like to point out that the values of the cation distribution have uncertainties of about 10%. For both cation distributions, the stoichiometry determined from AR-HAXPES was taken into account. We further point out that the cation distribution of the ${\mathrm{Co}}_{1.4}{\mathrm{Fe}}_{1.6}O_{4}$ film includes both the ${\mathrm{Co}}_{x}{\mathrm{Fe}}_{3-x}O_{4}$ phase and the Co–Fe oxide phase, which were reported in reference [18]. As discussed earlier, we expect that the effect due to the Co–Fe oxide phase is rather weak as the fraction of the ferrite phase in the film is considerably preponderant. Additionally, because the XA and XMCD spectra were fitted simultaneously and the crystallographic rock-salt phase should only contribute to the XAS signal, it should be contained within the limits of this fitting approach. Therefore, the determined cation distribution still mainly reflects the ${\mathrm{Co}}_{x}{\mathrm{Fe}}_{3-x}O_{4}$ phase. Consistently with our previous assumptions, both ${\mathrm{Co}}_{x}{\mathrm{Fe}}_{3-x}O_{4}$ films exhibit small amounts of ${\mathrm{Fe}}^{2+}$ and ${\mathrm{Co}}^{3+}$ cations, though the ${\mathrm{Co}}_{1.4}{\mathrm{Fe}}_{1.6}O_{4}$ film has a considerably larger amount. Since ${\mathrm{Fe}}^{3+}$ and ${\mathrm{Co}}^{2+}$ cations in both films still clearly predominate in terms of numbers, ${\mathrm{Fe}}^{2+}$ and ${\mathrm{Co}}^{3+}$ characteristic features do not contribute significantly in the (AR-)HAXPES and XA spectra.

In addition, the exact number of holes [$n_{h}\left({\mathrm{Co}}_{\mathrm{oct}}^{2+}\right)=2.89$, $n_{h}\left({\mathrm{Co}}_{\mathrm{tet}}^{2+}\right)=2.95$, $n_{h}\left({\mathrm{Co}}_{\mathrm{oct}}^{3+}\right)=3.83$, $n_{h}\left({\mathrm{Fe}}_{\mathrm{oct}}^{2+}\right)=3.82$, $n_{h}\left({\mathrm{Fe}}_{\mathrm{oct}}^{3+}\right)=4.89$, and $n_{h}\left({\mathrm{Fe}}_{\mathrm{tet}}^{3+}\right)=4.88$] of each film was extracted from the CTM calculations. As a consequence, we correct the total Co and Fe moments of the ${\mathrm{Co}}_{1.1}{\mathrm{Fe}}_{1.9}O_{4}$ film to $\left(1.37\pm 0.09\right)\;\mu_{B}$/Co ion and $\left(0.72\pm 0.05\right)\;\mu_{B}$/Fe ion, respectively. For the ${\mathrm{Co}}_{1.4}{\mathrm{Fe}}_{1.6}O_{4}$ film, we obtain a corrected total Co moment of $\left(0.90\pm 0.06\right)\;\mu_{B}$/Co ion and a corrected total Fe moment of $\left(0.74\pm 0.04\right)\;\mu_{B}$/Fe ion. Considering the stoichiometry determined by AR-HAXPES, we derive an overall magnetic moment of $\left(2.88\pm 0.28\right)\;\mu_{B}/f.u.$ for the ${\mathrm{Co}}_{1.1}{\mathrm{Fe}}_{1.9}O_{4}$ film and $\left(2.44\pm 0.23\right)\;\mu_{B}/f.u.$ for the ${\mathrm{Co}}_{1.4}{\mathrm{Fe}}_{1.6}O_{4}$ film at 300 K.

Based solely on the cation distribution determined from the CTM calculations and the theoretical spin moment of each individual cation, we obtain overall magnetic moments of $\left(3.67\pm 0.25\right)\;\mu_{B}/f.u.$ and $\left(3.47\pm 0.25\right)\;\mu_{B}/f.u.$ for the ${\mathrm{Co}}_{1.1}{\mathrm{Fe}}_{1.9}O_{4}$ film and ${\mathrm{Co}}_{1.4}{\mathrm{Fe}}_{1.6}O_{4}$ film, respectively. Since these estimates do not take into account thermal effects such as thermal agitation, they are not quite comparable with the experimentally derived values, and rather indicate the overall magnetic moments of both films at absolute zero, considering pure spin magnetic moments.

### 3.4. SQUID

**Figure 7:**
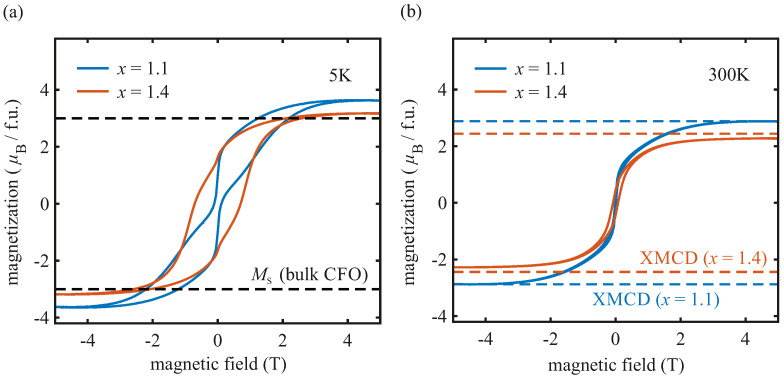
SQUID measurements for the ${\mathrm{Co}}_{x}{\mathrm{Fe}}_{3-x}O_{4}$ film with x=1.1 and x=1.4 at (**a**) 5 K and (**b**) 300 K. Dashed lines in (**a**) correspond to the values of the saturation magnetization $M_{s}$ for bulk CoFe${}_{2}$O${}_{4}$ [1] with complete inverse spinel structure, whereas dashed lines in (**b**) correspond to the overall magnetic moments of both films as derived from the XMCD analysis.

In order to study the integral magnetic properties of the formed cobalt ferrite films, complementary SQUID measurements at 5 K and 300 K were carried out. The measured magnetization was converted into the unit $\mu_{B}/f.u.$ for each magnetization curve. The external magnetic field was varied from −5 T to −5 T. Figure 7 shows the respective magnetization curves for both samples.

The magnetization curves taken at 5 K and 300 K show the typical hysteresis loops for ferro-/ferrimagnetic materials for each sample. For the ${\mathrm{Co}}_{x}{\mathrm{Fe}}_{3-x}O_{4}$ film with x=1.1, the saturation magnetization of $\left(3.63\pm 0.20\right)\;\mu_{B}/f.u.$ at 5 K exceeds the theoretical value of 3 $\mu_{B}/f.u.$ of stoichiometric bulk CoFe${}_{2}$O${}_{4}$ [1] (dashed lines). Enhanced saturation magnetization was also reported for thinner CoFe${}_{2}$O${}_{4}$ [35,67] and NiFe${}_{2}$O${}_{4}$ [68,69] films, and was attributed to a partial inverse cation distribution with divalent cations occupying both octahedral *B* sites and tetrahedral *A* sites. According to our CTM calculations of the XAS/XMCD measurements and the resulting overall magnetic moments based solely on the determined cation distribution and the spin moment of each individual cation, the enhanced saturation magnetization in our case can also be ascribed to the partial inverse spinel structure.

For the ${\mathrm{Co}}_{x}{\mathrm{Fe}}_{3-x}O_{4}$ film with x=1.4, the saturation magnetization of $\left(3.18\pm 0.20\right)\;\mu_{B}/f.u.$ at 5 K is lower compared to the ${\mathrm{Co}}_{x}{\mathrm{Fe}}_{3-x}O_{4}$ film with x=1.1. It has been shown that the amount of Co in ${\mathrm{Co}}_{x}{\mathrm{Fe}}_{3-x}O_{4}$ films strongly affects the net magnetization [15,16,50]. It was demonstrated that for Co concentrations of 1<x<2, the saturation magnetization of the ${\mathrm{Co}}_{x}{\mathrm{Fe}}_{3-x}O_{4}$ films decreases with increasing *x* [15,16]. The lower saturation magnetization with a higher concentration of Co cations in the cobalt ferrite film was also found to be related to a partial inverse spinel structure in combination with the presence of ${\mathrm{Co}}^{3+}$ cations at octahedral *B* sites in the low-spin state, both of which are in accordance with our XAS/XMCD results. Since the spin-related magnetic moment of ${\mathrm{Co}}^{3+}$ cations in its low-spin state is $0\;\mu_{B}/f.u.$ at octahedral *B* sites [cf. Figure 5a], an increase in the amount of ${\mathrm{Co}}^{3+}$ at these lattice sites would consequently reduce the net magnetization.

Additionally, the magnetization curve for ${\mathrm{Co}}_{1.1}{\mathrm{Fe}}_{1.9}O_{4}$ recorded at 5 K exhibits a large jump in the magnetization at low magnetic fields. A similar but weaker jump can also be seen for the ${\mathrm{Co}}_{1.4}{\mathrm{Fe}}_{1.6}O_{4}$ film. Similar observations have been reported in the literature for cobalt ferrite films deposited on several substrates—e.g., MgO(001) or SrTiO${}_{3}$(001) [14,35,70,71,72]—and their origins are still under discussion. Substrate induced strain effects [72], the presence of antiphase boundaries [14], and a second magnetic phase [35,71] are commonly considered to be the reason for this behavior of the magnetization.

One may assume that a very thin Fe${}_{3}$O${}_{4}$ interlayer between cobalt ferrite film and SrTiO${}_{3}$ substrate acts as a second magnetic phase, contributing also to the magnetization curves. Since the AR-HAXPES results indicate a homogeneous distribution of both Fe and Co cations [18], a very thin existing Fe${}_{3}$O${}_{4}$ film is unlikely but cannot be ruled out completely.

Furthermore, the crystallographic Co–Fe oxide rock-salt phase can be excluded as being responsible for this behavior of the magnetization. Since this second crystallographic phase is present exclusively in the ${\mathrm{Co}}_{1.4}{\mathrm{Fe}}_{1.6}O_{4}$ film, the magnetization curve of the ${\mathrm{Co}}_{1.4}{\mathrm{Fe}}_{1.6}O_{4}$ film should be the most pinched, assuming the second crystallographic phase is the culprit. However, the contrary is the case, and the magnetization curve of the ${\mathrm{Co}}_{1.1}{\mathrm{Fe}}_{1.9}O_{4}$ film is instead more pinched, which just does not contain this phase.

Rigato et al. [35] reported that a second ferrimagnetic phase, in their case, stemmed from the existence of pyramidal-shaped cobalt ferrite hut clusters, emerging from the surface, which dominate the magnetization curves more with decreasing film thickness of the cobalt ferrite film. According to the AFM results of both samples studied here (cf. Figure 3), it is possible that the jump in the magnetic moment in Figure 7 might also be attributed to this second magnetic phase due to pyramidal hut clusters at the surface of the ferrite film. Due to an increased volume fraction of the pyramidal-shaped cobalt ferrite hut clusters relative to the total film volume at lower film thicknesses, the second phase has a stronger contribution to the magnetization curves compared to cobalt ferrite films with higher film thicknesses (15% volume fraction for the ${\mathrm{Co}}_{1.1}{\mathrm{Fe}}_{1.9}O_{4}$ film and 10% volume fraction for the ${\mathrm{Co}}_{1.4}{\mathrm{Fe}}_{1.6}O_{4}$ film according to the AFM results). This may explain that the jump of the magnetic moment for the ${\mathrm{Co}}_{1.1}{\mathrm{Fe}}_{1.9}O_{4}$ film is more evident due to the larger fraction of the second hut cluster phase at the surface of the ferrite film, which is in accordance with the observations of Rigato et al. [35] and Coll et al. [14].

Compared to the saturation magnetization at 5 K the saturation magnetization at 300 K decreases from $\left(3.63\pm 0.20\right)\;\mu_{B}/f.u.$ to $\left(2.88\pm 0.20\right)\;\mu_{B}/f.u.$ for the ${\mathrm{Co}}_{1.1}{\mathrm{Fe}}_{1.9}O_{4}$ film and from $\left(3.18\pm 0.20\right)\;\mu_{B}/f.u.$ to $\left(2.28\pm 0.20\right)\;\mu_{B}/f.u.$ for the ${\mathrm{Co}}_{1.4}{\mathrm{Fe}}_{1.6}O_{4}$ film. Both saturation magnetizations at 300 K are in good agreement with the overall magnetic moments derived from the XAS/XMCD results also recorded at 300 K, confirming our analysis based on the CTM model. Comparing also the saturation magnetizations at 5 K of both samples with the overall magnetic moments based on the corresponding cation distribution and the theoretical spin moment of each cation species, the latter is larger than the measured value for the ${\mathrm{Co}}_{1.4}{\mathrm{Fe}}_{1.6}O_{4}$ film. The values of the ${\mathrm{Co}}_{1.1}{\mathrm{Fe}}_{1.9}O_{4}$ film agree nicely here as well. The deviation of the ${\mathrm{Co}}_{1.4}{\mathrm{Fe}}_{1.6}O_{4}$ film might very likely be related to the Co–Fe oxide precipitates mentioned earlier. In order to match the total magnetic moment of the film with the saturation magnetization of the SQUID results at 5 K, roughly 8% of all ${\mathrm{Co}}^{2+}$ cations from the cation distribution need to be assigned to the Co–Fe oxide precipitates (a pure CoO rock-salt phase was assumed for simplicity). This value is slightly smaller than obtained from our consideration before, assuming that precipitates are build if the Co content exceeds x=1.1.

Further, a temperature dependence of the saturation magnetization was also observed in other studies of cobalt ferrite thin films [14,37,71], cobalt ferrite nanoparticles [73,74,75], and cobalt ferrite single crystals [76]. In fact, the theoretical works of Bercoff and Bertorello [77] and Srivastava et al. [78] also showed a decrease of the magnetization of CFO with increasing temperatures, which is in accordance with the decrease in saturation magnetization of the ${\mathrm{Co}}_{x}{\mathrm{Fe}}_{3-x}O_{4}$ films presented in this case study. Consequently, we can most plausibly ascribe the decrease in saturation magnetization as observed in our Co ferrite films to the general dependence of magnetization on temperature.

## 4. Conclusions

We prepared two Co-rich ${\mathrm{Co}}_{x}{\mathrm{Fe}}_{3-x}O_{4}$ ultrathin films with stoichiometries x=1.1 and x=1.4 by means of intermixing epitaxial CoO/Fe${}_{3}$O${}_{4}$ bilayers on Nb-doped SrTiO${}_{3}$(001) via RSPE [18]. We performed a comprehensive analysis of the electronic and magnetic properties, employing both surface and bulk specific approaches. XAS and XMCD measurements across the Co $L_{2,3}$ and Fe $L_{2,3}$ edges in combination with charge-transfer multiplet calculations revealed the presence of ${\mathrm{Co}}^{3+}$ cations in the low-spin state at octahedral *B* sites and partial inverse spinel structures for both samples. A higher amount of low-spin ${\mathrm{Co}}^{3+}$ cations was found for the ${\mathrm{Co}}_{x}{\mathrm{Fe}}_{3-x}O_{4}$ film with higher Co content *x*, resulting in a decreased Co spin moment per ion and a lower overall magnetic moment. The SQUID measurements revealed enhanced saturation magnetizations at 5 K, which can be explained by the partial inverse spinel structure. A second soft magnetic phase in the magnetization loops might be explained by islands present on the surfaces of both samples, as observed by AFM measurements.

## Figures and Tables

**Table 1:** Orbital moment $m_{o}$, spin moment $m_{s}$, and total moment for the Co and Fe ions for the ${\mathrm{Co}}_{x}{\mathrm{Fe}}_{3-x}O_{4}$ films with Co contents of x=1.1 and x=1.4 determined from the Co XMCD and Fe XMCD spectra using the sum rules [41,42,43,44] and the sum rules’ correction factors as derived by Teramura et al. [45]. For comparison, the respective magnetic moments normalized to the number of holes ($n_{h}=3$ for the Co ions and $n_{h}=5$ for the Fe ions) are displayed underneath.

Co Content	Co Moment ($\mu_{B}$/Co ion)			Fe Moment ($\mu_{B}$/Fe ion)		
x	$m_{o}$	$m_{s}$	**total**	$m_{o}$	$m_{s}$	**total**
1.1	0.26±0.03	1.13±0.06	1.39±0.09	−0.04±0.01	0.80±0.04	0.76±0.05
1.4	0.16±0.02	0.72±0.04	0.88±0.06	0.01±0.01	0.76±0.04	0.77±0.05
x	$m_{o}/n_{h}$	$m_{s}/n_{h}$	**total**	$m_{o}/n_{h}$	$m_{s}/n_{h}$	**total**
1.1	0.09±0.01	0.38±0.02	0.47±0.03	−0.01±0.01	0.16±0.01	0.15±0.02
1.4	0.05±0.01	0.24±0.01	0.29±0.02	0.00±0.01	0.15±0.01	0.15±0.02

## Data Availability

The data presented in this study are available on reasonable request from the corresponding author.
